# *Clostridioides difficile* biofilms: A mechanism of persistence in the gut?

**DOI:** 10.1371/journal.ppat.1009348

**Published:** 2021-03-11

**Authors:** Lucy R. Frost, Jeffrey K. J. Cheng, Meera Unnikrishnan

**Affiliations:** Division of Biomedical Sciences, Warwick Medical School, University of Warwick, Coventry, United Kingdom; Geisel School of Medicine at Dartmouth, UNITED STATES

Biofilms are structured bacterial communities encased in an extracellular matrix. The structure and complexity of biofilms depend on the microorganism and the local environment [1,2]. Biofilms form on tissues and foreign implants during human infections and confer pathogens resistance to drugs and immune responses, making biofilm-associated infections extremely difficult to treat [1]. *Clostridioides difficile*, a major healthcare-associated gastrointestinal pathogen, causes *C*. *difficile* infection (CDI), which is associated with high rates of recurrence, especially in the elderly [3]. CDI is strongly associated with long-term antibiotic therapy, which results in disruption of the native gut microbiota. In recent years, *C*. *difficile* biofilms have been considered to be important for persistence of the bacterium in the gut and for recurrent infections. Here we review the current knowledge on *C*. *difficile* biofilms in the context of the gut environment and infection.

## *C*. *difficile* forms biofilms *in vitro*

Biofilm formation by *C*. *difficile* was first reported by Donelli and colleagues where they identified the role of polymicrobial biofilms in clogging of biliary stents using confocal and field emission scanning electron microscopy [4]. Soon after, biofilm formation by *C*. *difficile* strains of clinical origin (strains 630, R20291) on abiotic surfaces was reported, as quantitated by crystal violet staining [5,6]. Viable cell counts, as well as LIVE/DEAD viability staining showed that bacterial viability was higher in 1- to 3-day-old biofilms and decreased in 6-day-old biofilms [4–7]. *C*. *difficile* biofilms are multilayered, encased in a thick matrix composed of bacterial proteins, extracellular DNA (eDNA), and polysaccharide II; however, it is noteworthy that the composition and structure of biofilms are both time- and strain-dependent [5,7]. Numerous *C*. *difficile* factors which modulate biofilm formation have been identified, including pili, flagella proteins, the S-layer, Cwp84, quorum sensing, germination receptor SleC, and sporulation. Mutants deficient in stress-related proteins including the SOS response regulator, LexA, the RNA chaperone, Hfq, and the heat stress-associated chaperone, DnaK, have been associated with increased biofilm formation [8–10]. Interestingly, the toxins TcdA and TcdB were identified in the biofilm matrix of 3- and 6-day-old biofilms, suggesting that biofilms may play a role in *C*. *difficile* virulence [7]. Cyclic di-GMP (c-di-GMP) is thought to play an important role in the motile to sessile biofilm state shift through repression of flagellar synthesis and induction of pili [11]. In a recent global gene expression analysis of microfermentor biofilms, several genes controlled by the SinR-like regulators CD2214 and CD2215, including pilA1, were differentially expressed in biofilms, although pilA1 appeared to contribute to biofilm/aggregate formation only in c-di-GMP overexpressing strains [12]. Thus, *C*. *difficile* forms complex biofilms *in vitro* which involves multiple regulatory pathways and several virulence-associated proteins.

## Biofilms—A niche for *C*. *difficile* spore formation?

Spores are critical for transmission of CDI, and sporulation is a key pathway in *C*. *difficile* pathogenesis which is initiated under conditions of stress. Viable cell counts from biofilms formed by the clinically relevant strain, R20291, show that the majority of *C*. *difficile* cells are vegetative in 3-day-old biofilms. However, the number of spores increased over time, with spores forming the majority of cells in 6-day-old biofilms [6,7]. A sporulation-deficient *C*. *difficile* strain lacking Spo0A, a master transcriptional regulator which induces the sporulation pathway upon phosphorylation, formed significantly reduced biofilms compared to the wild type [5,6]. The *spo0A* mutant biofilms were easily detached and had significantly less resistance to oxygen stress than the wild type [6]. Together, these findings suggest that biofilm formation may be regulated by Spo0A, indicating an intriguing link between spore formation and biofilms. Furthermore, Semenyuk and colleagues found that spores from biofilm cultures had a reduced germination efficiency compared to conventionally cultured spores [7]. Differences observed in the exosporium structure of spores from planktonic and biofilm cultures may contribute to increased thermotolerance and reduced germination efficiency in biofilm-derived spores [7,13,14]. Collectively, although we lack direct evidence from infection, these findings suggest that *C*. *difficile* biofilms can serve as a niche for generating modified spores, which favour maintenance of a dormant population, aiding bacterial persistence and disease recurrence.

## *C*. *difficile* biofilms protect from antibiotics

Biofilm-associated antibiotic tolerance is the result of a myriad of factors, including the type of antibiotic, bacterial species, biofilm stage, and availability of resources [1]. The ability to form biofilms allows *C*. *difficile* to resist antibiotics and oxidative stresses [2,5,6]. When *C*. *difficile* is exposed to varying levels of vancomycin, a drug commonly used to treat CDI, bacteria survived better and displayed resistance in a biofilm compared to planktonic culture [5]. In a triple-stage human gut model, vancomycin treatment reduced planktonic *C*. *difficile* to below the detection limit, while the biofilm population remained unchanged [15]. Utilising a colony biofilm model, treatment of *C*. *difficile* biofilms grown on black polycarbonate membranes with 100x minimum inhibitory concentration (MIC) of metronidazole, another drug used to treat CDI, resulted in a significant decrease in bacterial numbers compared to vancomycin at 100x MIC [16]. However, for both vancomycin and metronidazole, the biofilms only delayed killing and neither were successful in reducing viable spores [16]. Fidaxomicin, a newer antibiotic that is effective for recurrent infections, at 25x MIC, was able to reduce biofilm bacterial and spore viability by approximately 2.5- and 1.5-fold, respectively. Surotomycin, a cyclic lipopeptide, showed similar abilities, yielding a 3-fold reduction of vegetative cells and 1.5-fold reduction in spore viability, suggesting a quicker penetration and greater disruptive ability of fluorescently labelled fidaxomicin compared to surotomycin [16]. A recent larger scale study which assayed antimicrobials including thuricin CD, tigecycline, vancomycin, teicoplanin, rifampicin, and nitazoxanide, against a variety of *C*. *difficile* strains in sessile and planktonic modes, showed that pairwise combinations of antimicrobials were more effective than single antibiotic treatments against R20291 biofilms, except nitazoxanamide, whose potency was reduced when combined with thuricin CD. Sensitivity to drugs or drug combinations was shown to be strain-dependent, with strains producing varied levels of biofilms *in vitro* [17].

With regard to the mechanisms underlying antibiotic resistance, the dense biofilm matrix can act as a physical barrier, providing resistance to antimicrobial penetration, and disrupted biofilms were more susceptible to antibiotics compared to intact biofilms [1,5]. Paradoxically, subinhibitory concentrations of metronidazole and vancomycin induced biofilm formation and seemingly reduced antibiotic susceptibility [5,18]. Therefore, it is possible that low levels of antibiotics could induce *C*. *difficile* biofilm production, thus promoting persistence and recurrence of infection.

## *C*. *difficile* interactions with the microbiota

*C*. *difficile* establishes itself in the intestine only when the native gut microbiota is altered, usually by treatment with broad spectrum antibiotics like fluoroquinolones. Colonisation resistance provided by intestinal bacteria prevents *C*. *difficile* from colonising through different mechanisms, including generation of nutritional niches, production of antimicrobial peptides, metabolites, and quorum sensing [3]. Commensals like *Clostridium scindens* have been associated with resistance to infection through production of secondary bile acids like deoxycholate, which prevent *C*. *difficile* growth [19]. *Bacteroides* spp. can prevent *C*. *difficile* growth, both *in vitro* and *in vivo*; interestingly, *B*. *fragilis* appears to inhibit *C*. *difficile* only when in close contact within mixed biofilms, through an autoinducer-2–mediated mechanism [20]. In a culturomics study, 66 species isolated from microbiota were found to inhibit *C*. *difficile*. When bacterial species combinations were tested for their inhibitory effects, species composition and blend size were found to be important for *C*. *difficile* inhibition, suggesting that bacterial interactions play a role in the inhibitory effects [21].

However, recent research has indicated that *C*. *difficile* also closely interacts with the gut microbiota during colonisation. A recent study showed that deoxycholate from the gut commensal species, *C*. *scindens*, induced *C*. *difficile* biofilm formation, indicating that deoxycholate effects on *C*. *difficile* may be concentration-dependent [22]. *C*. *difficile* and the commensal *Fusobacterium nucleatum* were reported to coaggregate *in vitro*, increasing biofilm formation and extracellular polysaccharide production [23]. Studies employing a triple stage human gut chemostat model have shown that *C*. *difficile* was present within sessile microbiota communities from faecal emulsions [15]; spores were predominantly found in these communities, which appeared to germinate over time. In one of the early *in vivo* studies to examine the presence of *C*. *difficile* multicellular communities during infection, the Driks laboratory showed that low numbers of *C*. *difficile* were present within mixed communities containing Bacteroidetes and Firmicutes species on the outer mucus layer of the gut in a *C*. *difficile* murine infection model [24]. Thus, the data point to *C*. *difficile* forming adherent communities in close association with the commensal microbiota species.

## *C*. *difficile* biofilms during infection

During infection, biofilms may serve as reservoirs of *C*. *difficile*, which allow bacteria to persist in the gut in the presence of antibiotic therapy, potentially reestablishing infections and resulting in recurrent disease. Nevertheless, a direct role for biofilms in the recurrence of *C*. *difficile* infection is yet to be demonstrated. Bacterial factors that are necessary for biofilm formation are essential for colonisation and virulence in many gastrointestinal pathogens. Some *C*. *difficile* surface and regulatory factors key for biofilm formation such as flagella, pili, and Spo0A also have colonisation defects in murine models of infection, indicating that adherence to gut surfaces is important during infection [3]. However, a role for these factors in biofilm formation during infection has not been formally demonstrated.

**Fig 1:**
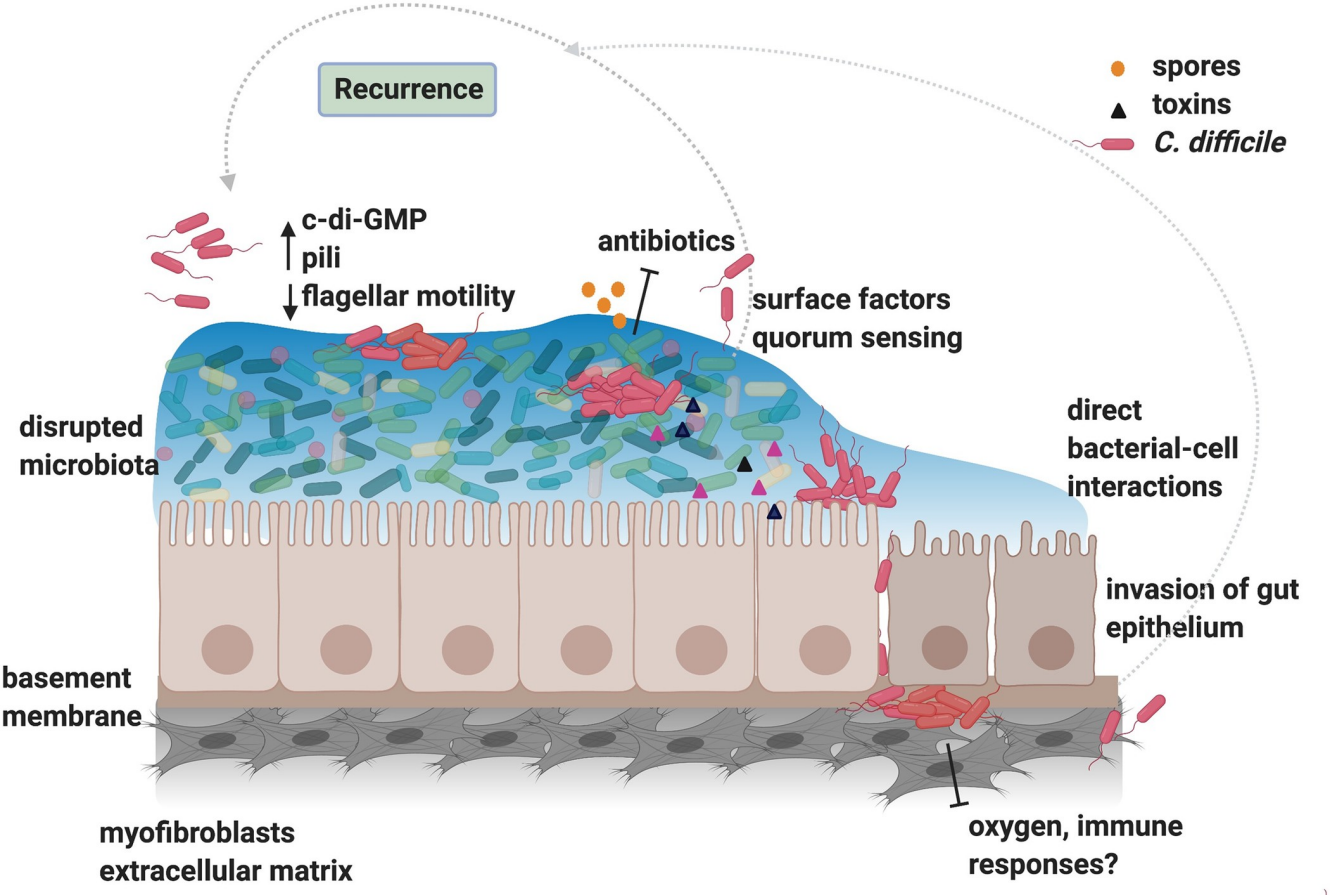
A model of *C*. *difficile* biofilms during infection. *C*. *difficile* initially attaches to the mucosal layers in the gut, when the native gut microbiota is disrupted by broad spectrum antibiotics. Increased c-di-GMP levels resulting in decreased bacterial motility enables attachment and establishment of microaggregates or communities. These communities could exist as single species or in close association with the gut microbiota, serve as a niche for production of spores and toxins (toxins A, B, and binary *C*. *difficile* toxin), and provide protection from oral antibiotics using for treatment (e.g., vancomycin, metronidazole) in the lumen. Surface factors (e.g., pili, flagella, S-layer), quorum sensing (e.g., LuxS), and regulators (e.g., Spo0A, CD630_2214) control biofilm/aggregate formation. Direct bacterial interactions of *C*. *difficile* and action of toxins trigger cell death and disruption of epithelial barrier, allowing bacteria to penetrate the epithelial cell layer to the underlying basement membrane and myofibroblasts. *C*. *difficile* may form communities in underlying tissue which may protect bacteria from oxygen and immune responses. Bacterial communities in the gut mucosa may allow bacterial persistence, and under conducive conditions, bacteria may be dispersed, leading to reseeding and recurrence of infection. *Image created with BioRender.com*.

A general challenge with defining the role of biofilms during infection by gastrointestinal pathogens is the visualisation of biofilm communities, which is confounded by the microbiota lining the gut. *C*. *difficile* microcolonies and filaments were observed on epithelial cells during infection in an *in vitro* gut infection model [25], and biofilm-like *C*. *difficile* cell aggregates have also been reported from hamster and murine infection models [26,27]. A recent study demonstrated that *C*. *difficile* forms mono-species biofilm communities in gnotobiotic mice [2]. Different *C*. *difficile* strains were reported to colonise the murine gut similarly with bacterial aggregates associated to the mucus layer rather than with the epithelial cells [2]. Although this study suggests that *C*. *difficile* is capable of building communities *in vivo*, the formation of such communities in the context of the native microbiota needs further study. While communities likely form in conjunction with the microbiota, communities may also form within deeper cell layers during invasive infection. Based on our current knowledge, we propose a model for how *C*. *difficile* biofilms may form during infection (Fig 1).

## Future perspectives

*C*. *difficile* biofilm communities are likely critical in recurrent CDI. Although we have a good understanding of *C*. *difficile* biofilm formation and regulation *in vitro*, several questions regarding the relevance of such biofilm communities in bacterial persistence and the bacterial and host factors regulating their formation *in vivo* remain unanswered. New visualisation tools and cutting-edge gut infection models for *C*. *difficile* combined with further studies on clinical samples will likely provide better insight into the role of *C*. *difficile* biofilms in CDI.

## References

[1] Hall CW, Mah TF. Molecular mechanisms of biofilm-based antibiotic resistance and tolerance in pathogenic bacteria. FEMS Microbiol Rev. 2017;41:276–301. 10.1093/femsre/fux010 28369412

[2] Soavelomandroso AP, Gaudin F, Hoys S, Nicolas V, Vedantam G, Janoir C, et al. Biofilm structures in a mono-associated mouse model of Clostridium difficile infection. Front Microbiol. 2017;8:1–10. 10.3389/fmicb.2017.00001 29118745 PMC5661025

[3] Abt MC, McKenney PT, Pamer EG. *Clostridium difficile* colitis: pathogenesis and host defence. Nat Rev Microbiol. 2016;14:609–20. 10.1038/nrmicro.2016.108 27573580 PMC5109054

[4] Donelli G, Vuotto C, Cardines R, Mastrantonio P. Biofilm-growing intestinal anaerobic bacteria. Immunol Med Microbiol. 2012;65:318–25. 10.1111/j.1574-695X.2012.00962.x 22444687

[5] Dapa T, Leuzzi R, Ng YK, Baban ST, Adamo R, Kuehne SA, et al. Multiple factors modulate biofilm formation by the anaerobic pathogen *Clostridium difficile*. J Bacteriol. 2013;195:545–55. 10.1128/JB.01980-12 23175653 PMC3554014

[6] Dawson LF, Valiente E, Faulds-Pain A, Donahue EH, Wren BW, Popoff MR. Characterisation of *Clostridium difficile* Biofilm Formation, a Role for Spo0A. PLoS ONE. 2012;7. 10.1371/journal.pone.0050527 23236376 PMC3517584

[7] Semenyuk EG, Laning ML, Foley J, Johnston PF, Knight KL, Gerding DN, et al. Spore Formation and Toxin Production in *Clostridium difficile* Biofilms. PLoS ONE. 2014;9:e87757. 10.1371/journal.pone.0087757 24498186 PMC3907560

[8] Jain S, Smyth D, O’Hagan BMG, Heap JT, McMullan G, Minton NP, et al. Inactivation of the dnaK gene in *Clostridium difficile* 630 Δerm yields a temperature-sensitive phenotype and increases biofilm-forming ability. Sci Rep. 2017;7:1–13. 10.1038/s41598-016-0028-x 29235503 PMC5727486

[9] Boudry P, Gracia C, Monot M, Caillet J, Saujet L, Hajnsdorf E, et al. Pleiotropic role of the RNA chaperone protein Hfq in the human pathogen *Clostridium difficile*. J Bacteriol. 2014;196:3234–48. 10.1128/JB.01923-14 24982306 PMC4135688

[10] Walter BM, Cartman ST, Minton NP, Butala M, Rupnik M. The SOS response master regulator LexA is associated with sporulation, motility and biofilm formation in *Clostridium difficile*. PLoS ONE. 2015;10:1–17. 10.1371/journal.pone.0144763 26682547 PMC4689574

[11] Purcell EB, McKee RW, McBride SM, Waters CM, Tamayo R. Cyclic diguanylate inversely regulates motility and aggregation in *Clostridium difficile*. J Bacteriol. 2012;194:3307–16. 10.1128/JB.00100-12 22522894 PMC3434733

[12] Poquet I, Saujet L, Canette A, Monot M, Mihajlovic J, Ghigo JM, et al. Clostridium difficile Biofilm: Remodeling metabolism and cell surface to build a sparse and heterogeneously aggregated architecture. Front Microbiol. 2018;9:1–20. 10.3389/fmicb.2018.00001 30258415 PMC6143707

[13] Pizarro-Guajardo M, Calderón-Romero P, Paredes-Sabja D. Ultrastructure variability of the exosporium layer of *Clostridium difficile* spores from sporulating cultures and biofilms. Appl Environ Microbiol. 2016;82:5892–8. 10.1128/AEM.01463-16 27474709 PMC5038037

[14] Pickering DS, Wilcox MH, Chilton CH. Biofilm-derived spores of *Clostridioides (Clostridium) difficile* exhibit increased thermotolerance compared to planktonic spores. Anaerobe. 2018;54:169–71. 10.1016/j.anaerobe.2018.10.003 30292821

[15] Crowther GS, Chilton CH, Todhunter SL, Nicholson S, Freeman J, Baines SD, et al. Development and Validation of a Chemostat Gut Model To Study Both Planktonic and Biofilm Modes of Growth of *Clostridium difficile* and Human Microbiota. PLoS ONE. 2014;9. 10.1371/journal.pone.0088396 24516647 PMC3916432

[16] James GA, Chesnel L, Boegli L. Pulcini E de L, Fisher S, Stewart PS. Analysis of *Clostridium difficile* biofilms: Imaging and antimicrobial treatment. J Antimicrob Chemother. 2018;73:102–8. 10.1093/jac/dkx353 29029221

[17] Mathur H, Rea MC, Cotter PD, Hill C, Ross RP. The efficacy of thuricin CD, tigecycline, vancomycin, teicoplanin, rifampicin and nitazoxanide, independently and in paired combinations against *Clostridium difficile* biofilms and planktonic cells. Gut Pathog. 2016;8:1–10. 10.1186/s13099-015-0083-z 27257437 PMC4890490

[18] Vuotto C, Moura I, Barbanti F, Donelli G, Spigaglia P. Subinhibitory concentrations of metronidazole increase biofilm formation in *Clostridium difficile* strains. Pathog Dis. 2016;74:1–7. 10.1093/femspd/ftv114 26656887

[19] Buffie CG, Bucci V, Stein RR, McKenney PT, Ling L, Gobourne A, et al. Precision microbiome reconstitution restores bile acid mediated resistance to *Clostridium difficile*. Nature. 2015;517:205–8. 10.1038/nature13828 25337874 PMC4354891

[20] Slater RT, Frost LR, Jossi S, Millard AD, Unnikrishnan M. *Clostridioides difficile* LuxS mediates inter-bacterial interactions within biofilms. Sci Rep. 2019;9:9903. 10.1038/s41598-019-46143-6 31289293 PMC6616478

[21] Ghimire S, Roy C, Wongkuna S, Antony L, Maji A, Keena C. Identification of *Clostridioides difficile*-Inhibiting Gut Commensals Using Culturomics, Phenotyping and Combinatorial Community Assembly. mSphere. 2020;5:1–19. 10.1128/mSystems.00620-19 32019832 PMC7002114

[22] Dubois T, Tremblay YDN, Briandet R. Dupuy B. A microbiota-generated bile salt induces biofilm formation in Clostridium difficile. NPJ Biofilms Microbes. 2019;5:1–12. 10.1038/s41522-019-0087-4 31098293 PMC6509328

[23] Engevik M, Danhof HA, Auchtung J, Endres BT, Ruan W, Bassères E, et al. *Fusobacterium nucleatum* adheres to *Clostridioides difficile* via the RadD adhesin to enhance biofilm formation in intestinal mucus. Gastroenterology. 2020; 104743. Available: 10.1053/j.gastro.2020.11.034 33227279 PMC7956072

[24] Semenyuk EG, Poroyko VA, Johnston PF, Jones SE, Knight KL, Gerding DN, et al. Analysis of bacterial communities during *Clostridium difficile* infection in the mouse. Infect Immun. 2015;83:4383–91. 10.1128/IAI.00145-15 26324536 PMC4598419

[25] Anonye BO, Hassall J, Patient J, Detamornrat U, Aladdad AM, Schüller S, et al. Probing *Clostridium difficile* Infection in Complex Human Gut Cellular Models. Front Microbiol. 2019;10:1–15. 10.3389/fmicb.2019.00001 31114553 PMC6503005

[26] Lawley TD, Clare S, Walker AW, Goulding D, Stabler RA, Croucher N, et al. Antibiotic treatment of *Clostridium difficile* carrier mice triggers a supershedder state, spore-mediated transmission, and severe disease in immunocompromised hosts. Infect Immun. 2009;77:3661–9. 10.1128/IAI.00558-09 19564382 PMC2737984

[27] Buckley AM, Spencer J, Candlish D, Irvine JJ, Douce GR. Infection of hamsters with the UK *Clostridium difficile* ribotype 027 outbreak strain R20291. J Med Microbiol. 2011;60:1174–80. 10.1099/jmm.0.028514-0 21330415 PMC3167879

